# From Crisis to Cure: The Resilient Recovery of a Patient With Tetanus

**DOI:** 10.7759/cureus.75551

**Published:** 2024-12-11

**Authors:** Elangovan Raman, Naveenkumar Nallathambi, Gautham Raghuthaman, Sathyanarayanan MM, Samuel Dinesh A

**Affiliations:** 1 Internal Medicine, Madras Medical College and Rajiv Gandhi Government General Hospital, Chennai, IND

**Keywords:** clostridium bacilli, rhabdomyolysis, tetanus, trismus, vaccination

## Abstract

Tetanus is a severe neurological condition triggered by the toxin of *Clostridium tetani*, resulting in extreme muscle stiffness and spasms. Although vaccination can prevent it, without treatment, tetanus carries a high risk of death due to respiratory failure and autonomic disturbances. This case report describes a 24-year-old Indian male who developed tetanus after branding (a traditional procedure for jaundice in rural India) on his wrist. He presented with neck stiffness, hypertonia, and limb pain. Based on the presentation and poor immunization history, he was diagnosed with tetanus. He was managed with IV fluids, antibiotics, tetanus immunoglobulin, tetanus toxoid, and diazepam. This case underscores the importance of early recognition, proper wound care, and immunization for tetanus prevention.

## Introduction

Tetanus is a severe and potentially fatal disease caused by the neurotoxin tetanospasmin produced by *Clostridium tetani*. The disease is characterized by progressive muscle rigidity and spasms, which can lead to significant morbidity and mortality if not promptly treated. Despite advances in vaccination and public health measures, tetanus remains a critical concern in developing regions, where conditions such as poor wound care and inadequate immunization coverage persist [1]. Tetanus is more prevalent in developing countries like India, attributed to the humid climate, warm temperatures, and high-risk populations, including those who are immunodeficient, elderly with waning immunity, diabetic individuals, and those with no immunization history.

The disease commonly manifests with trismus (lockjaw), muscle pain, stiffness, and difficulty swallowing. In severe cases, it can progress to generalized muscle spasms and autonomic dysfunction. Tetanus is often associated with injuries or wounds, particularly those involving soil or animal feces, which are common in rural and agricultural settings [2]. Branding is a traditional indigenous healing practice. In ancient India, people used thermal cautery and branding therapy. Traditional healers used it to treat joints, spine and nerve disorders, jaundice, abdominal pain, breathing difficulty, and paralysis. The providers commonly applied hot metal rods, heated nails, wires, incense sticks, and hot bangles on the face, forehead, abdomen, and chest wall [3]. Such dangerous practices are more prevalent in rural India, especially among indigenous communities, and at times cause significant morbidity and delay in receiving appropriate medical care.

This case report details the clinical presentation and management of a 24-year-old male from a rural area in India who developed tetanus following a branding injury. The patient’s unvaccinated status and his occupational exposure to soil contributed to his susceptibility. The report highlights the critical importance of timely diagnosis, comprehensive treatment, and preventive measures such as vaccination and proper wound care. It aims to underscore the ongoing challenges in managing tetanus in settings with limited resources and to reinforce the necessity of effective public health interventions to prevent and control this life-threatening condition.

## Case presentation

**Figure 1:**
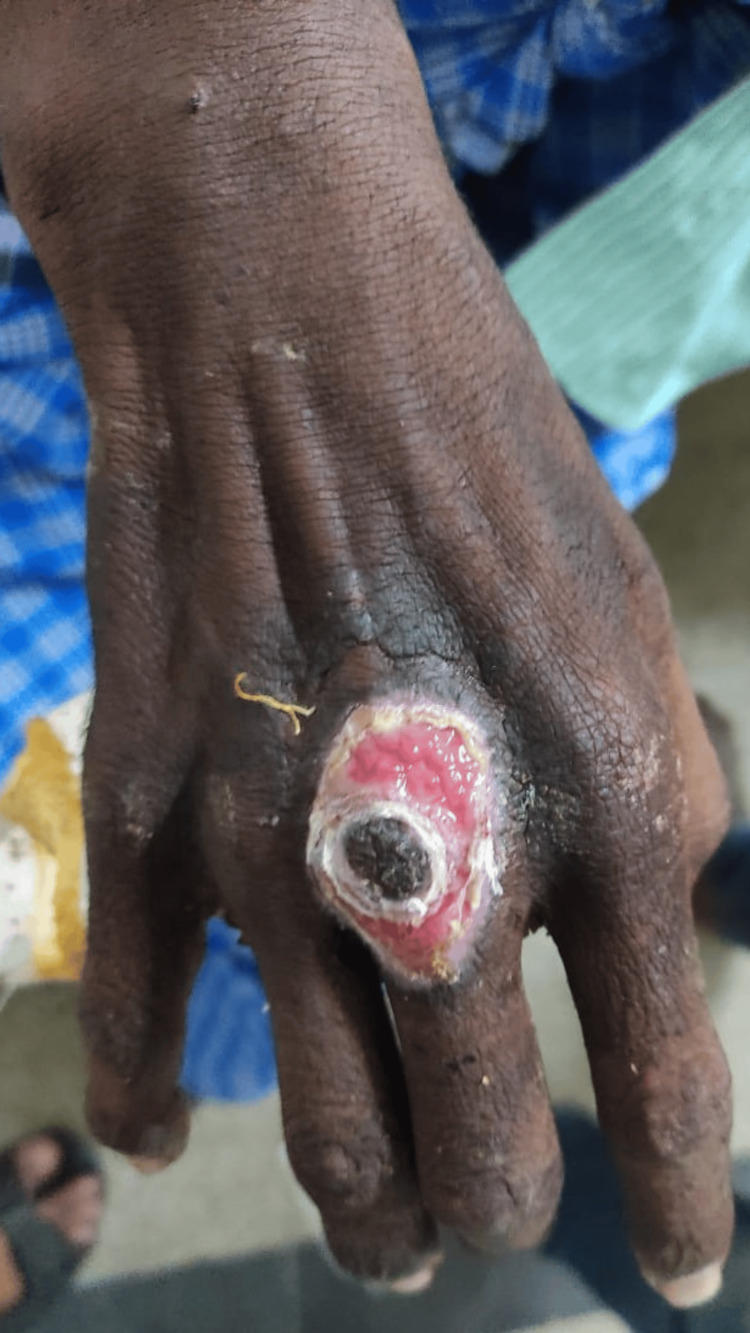
A 4x5 cm open wound due to branding on the right dorsum of the hand without granulation tissue, slough, or bleeding

A 24-year-old male farmer by occupation from a rural village in India had experienced jaundice two months prior, for which he underwent branding (a native Indian practice involving a third-degree burn) on the right wrist. Two months following the procedure, he developed left upper limb pain and neck stiffness. He took analgesics, which provided no relief. He progressed to develop difficulty opening his mouth upon waking up and difficulty eating with drooling of saliva. No other signs of meningeal irritation were noted at the time. He was not immunized appropriately and had no other drug intake other than analgesics. No similar episodes in the past and no significant family history. Upon examination, the patient was conscious, oriented, afebrile, and had no tachypnea or pedal edema. Vital examination was stable with a blood pressure of 120/90 mmHg, a pulse rate of 99/min, and an oxygen saturation of 99% in room air. Local examination identified a 4x5 cm open wound on the right dorsum of the hand without granulation tissue, slough, or bleeding (Figure 1).

**Figure 2:**
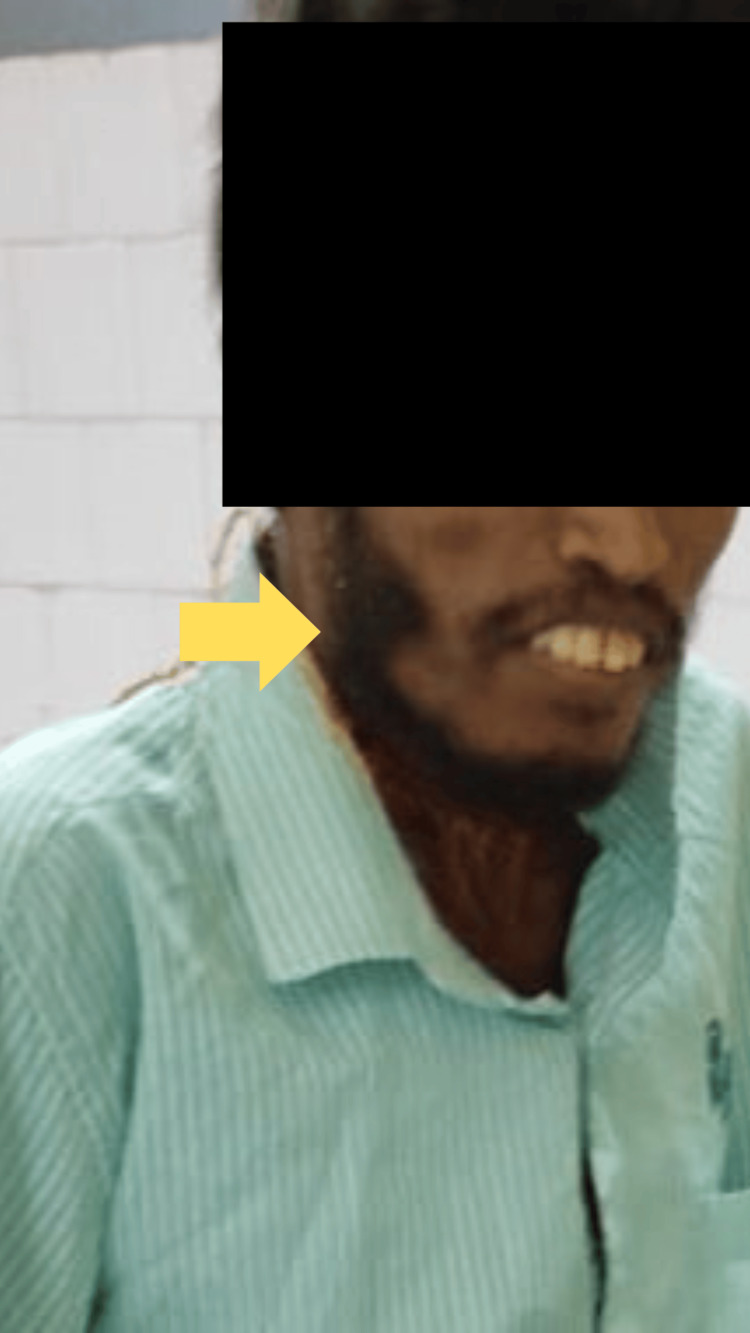
Trismus - lock jaw

Neurological examination showed neck stiffness, generalized hypertonia, opisthotonus, and difficulty opening the mouth (trismus) (Figure 2).

The cardiovascular and respiratory examinations were normal. The abdomen was soft and non-tender, with no organomegaly. A clinical diagnosis of tetanus was made on the grounds of poor immunization, exposure to soil, and the recent branding procedure. The basic investigation showed normal hemograms and elevated bilirubin, urea, creatinine, and creatine phosphokinase, as seen in Table 1.

**Table 1:** Basic investigation showed normal hemogram, elevated bilirubin, urea, creatinine, creatine phosphokinase, etc.

Lab parameters	Patient value	Reference values
Haemoglobin	13.2 g/dL	11.5–15.5 g/dL
White cell count	11,000/mm3	5,000–10,000/mm3
Platelet	278x10^9^/L	150 to 400 x 10^9^/L
Sodium	141mEq/L	136-145mE/L
Potassium	5.2 mmol/L	3.5-5 mmol/L
Uric acid	5 mg/dL	3.5-7.2 mg/dL
Phosphorus	3.2 mg/dL	2.8 to 4.5 mg/dL
Urea	62 mg/dL	8–24 mg/dL
Creatinine	1 mg/dL	0.7-1.3 mg/dL
Creatine phosphokinase	1200 mcg/L	10-120 mcg/L

His disease course was complicated by rhabdomyolysis with acute kidney injury. He was started on inj. tetanus immunoglobulin 5000 units intramuscularly and Inj. tetanus toxoid 0.5 ml IM, IV fluids, and diazepam. Antibiotics given were IV crystalline penicillin 10 million units for 10 days and IV metronidazole 500 mg for 19 days. He was monitored closely for electrolyte imbalance, urine output, and autonomic disturbance. The patient responded positively to treatment, showing signs of reduced hypertonia, trismus, and improved limb strength. This clinical improvement is supported by laboratory results, demonstrating a reduction in relevant values. Initially requiring support, the patient regained the ability to sit, walk, and tolerate oral feeds. On follow-up, three weeks after discharge, he demonstrated complete recovery.

## Discussion

Tetanus is a non-communicable vaccine-preventable disease. *Clostridium tetani* is a gram-positive, spore-forming, motile, anaerobic bacillus. The most common source of environmental exposure to *Clostridium tetani* bacilli and spores is soil. It is also found host in both herbivorous and carnivorous animals who excrete it in their feces. Fecal carriage has been reported in 10% to 20% of horses and 25% to 30% of dogs and guinea pigs. Fecal specimens from several other species, including sheep, cattle, and small mammals, also contained *Clostridium tetani*. They are strict anaerobes growing between optimal temperatures of 33° to 37°C [4]. It produces a neurotoxin, tetanospasmin, which binds to presynaptic alpha motor nerve terminals (polysialogangliosides), undergoes internalization, and is retrogradely transported along axons to the motor nuclei of cranial nerves or ventral horns of the spinal cord. Subsequently, it translocates across the synapse of GABA and glycine inhibitory interneuron terminals, inhibiting neurotransmitter release [5]. This results in unopposed excitatory stimuli on the skeletal muscles, leading to spastic paralysis and autonomic dysfunction. The incubation period typically lasts between seven and 10 days, although it can last up to 60 days. The degree of symptoms is correlated with the distance to the central nervous system from the site of inoculation; shorter incubation times are linked to more severe symptoms [6].

Tetanus manifests in four types: generalized (descending spastic paralysis with exaggerated deep tendon reflexes), localized (spastic paralysis affecting a specific limb), cephalic (neck stiffness, retracted eyelids, deviated gaze, risus sardonicus, and peculiar flaccid paralysis of cranial nerves), and neonatal [7]. Generalized tetanus is more common, affecting muscles near the wound site, while cephalic tetanus is less common and presents symptoms like dysphagia, trismus, and risus sardonicus. Localized tetanus affecting muscle groups beyond the typical presentation is exceptionally rare, with scant documented instances in published literature frequently mimicking alternative pathological conditions. Opisthotonus is relatively rare. It is a sign that may be present in a wide variety of disorders. It can affect infants (e.g., neonatal tetanus), children, or adults. There is no gender predilection. It is indeed difficult to diagnose in the younger age group. As opisthotonus is neither a disorder nor a disease, the incidence and prevalence are not reported [8].

The major risk factors of neonatal tetanus are unvaccinated mothers, unsafe home delivery care, prior children with a history of tetanus, and traditional practices like cow dung application on the umbilical stump [9].

The management of tetanus involves passive immunization with tetanus immunoglobulin along with antibiotics and active immunization with tetanus toxoid. Debridement of the wound will control the source of toxin production. Metronidazole has been demonstrated to decrease the disease's progression, despite the fact that toxins are the primary cause of the illness. It has also been demonstrated that metronidazole reduces mortality. Penicillin was used in the management, and it was later found to worsen the disease with synergistic action with tetanospasmin [10]. Other pharmacotherapy involves using muscle relaxants like baclofen and benzodiazepines to decrease anxiety [11]. Supportive measures like bedsore prevention, mechanical ventilation, tracheostomy, and a high-calorie diet compensate for the increased catabolic state. The Centers for Disease Control and Prevention guidelines outline specific recommendations for administering tetanus prophylaxis based on the history of tetanus toxoid-containing vaccines and the nature of the wound, as seen in Table 2 [12]. It can be prevented by vaccination following any wound.

**Table 2:** CDC guidelines outline specific recommendations for administering tetanus prophylaxis based on the history of tetanus toxoid-containing vaccines and the nature of the wound CDC: Centers for Disease Control and Prevention [12]

Vaccination status	Clean wound	Dirty or severe wounds
Unknown <3 doses	Tetanus vaccine	Tetanus vaccine with tetanus immunoglobulin
Completed >3 doses	Tetanus vaccine only if the last dose >10 years ago	Tetanus vaccine only if last dose > 5 years ago. Tetanus Immunoglobulin not needed

The major complications associated with tetanus include respiratory failure, which can occur due to laryngeal spasms leading to airway obstruction and difficulty breathing. Sudden cardiac arrest is another critical complication resulting from autonomic disturbances that cause rapid fluctuations in blood pressure, tachycardia, or bradycardia, potentially leading to heart block. Acute renal failure may develop as a consequence of rhabdomyolysis induced by the toxins produced by *Clostridium tetani*. Additionally, tetanus can cause pressure sores, contractures, and residual weakness.

Kakou et al. reported that 38% of patients acquired infection via limb injuries, whereas only 2% had abdominal wounds. Notably, 37 patients (82%) achieved a cure, with five cases (11%) experiencing sequelae, and a total of seven fatalities were recorded (16%). All individuals in this series lacked sufficient immune prophylaxis, and the primary risk factor identified was the secondary generalization of tetanus [13]. The WHO estimated a worldwide death in 1997 to be around 275,000, with an improvement to 14,132 in 2011. There is greater mortality variation in developing countries than in developed countries due to the accessibility of resources like mechanical ventilation and early recognition. Deliveries done with unclean hands or surfaces can transmit tetanus in newborns. In 2018, around 25,000 newborns died of neonatal tetanus, which is 97% compared to 1988 (787,000 deaths). There is an increased risk of tetanus in adolescent males who undergo circumcision due to waning immunity and limited accessibility to booster doses [14]. IV drug abusers are also at an increased risk of developing tetanus [15].

The differential for tetanus is neuroleptic malignant syndrome, which occurs following an exposure to an antipsychotic, which was not noted in our case. Patients with malignant neuroleptic syndrome can present with striking symptoms of autonomic instability and muscular rigidity. However, the presence of fever, altered mental status, and recent receipt of an agent with a propensity to cause this complication usually makes the distinction from tetanus relatively easy. Another mimic is strychnine poisoning. It may produce a clinical syndrome similar to tetanus. Supportive care for both conditions is critical; thus, the initial treatment of both conditions is identical. Assays of blood, urine, and tissue for strychnine can be performed in special reference laboratories. Such tests should be obtained when there is any suspicion of accidental or intentional poisoning, when a typical history of an antecedent injury or infection for tetanus is lacking, or when the patient has been adequately immunized for tetanus [16]. This case highlights the importance of vaccination, prompt medical intervention, and avoiding high-risk traditional treatment methods.

**Table 3:** Comparative table showing similarities and differences in the presentation of tetanus and neuroleptic malignant syndrome [16]

	Tetanus	Neuroleptic malignant syndrome
Trigger	Injury	Dopamine antagonist (antipsychotic drugs)
Rigidity	Profound	Profound
Autonomic instability	Yes	Yes
Mental status change	No	Very common
Antidote	Tetanus toxoid, immunoglobulin	Bromocriptine

## Conclusions

Effective tetanus management and prevention hinge on a comprehensive vaccination strategy and timely medical intervention. Vaccination remains the cornerstone of tetanus prevention, offering robust protection against this potentially life-threatening infection.

Routine immunization schedules for children, combined with booster doses for adults every ten years, are crucial in maintaining immunity and reducing the incidence of tetanus. For individuals with wounds or injuries, prompt medical evaluation and, if necessary, booster administration are essential to prevent infection. Additionally, public awareness and education about the importance of vaccination and proper wound care play a significant role in controlling tetanus. By adhering to these preventive measures and ensuring widespread vaccination coverage, we can significantly diminish the risk of tetanus and protect public health.

## References

[1] Rhinesmith E, Fu L (2018). Tetanus disease, treatment, management. Pediatr Rev.

[2] Bleck TP (1991). Tetanus: pathophysiology, management, and prophylaxis. Dis Mon.

[3] Jena S, Sahoo KC, Modak B, Epari V, Satpathy SK, Kaur H, Pati S (2022). An ethnographic approach to understand cultural perspectives of tribes on branding practice for sick children in Odisha, India. Indian J Med Res.

[4] Roper MH, Wassilak SG, Tiwari TS, Orenstein WA (2013). Vaccines (sixth edition). Tetanus toxoid.

[5] Yeh FL, Dong M, Yao J, Tepp WH, Lin G, Johnson EA, Chapman ER (2010). SV2 mediates entry of tetanus neurotoxin into central neurons. PLoS Pathog.

[6] Fan Z, Zhao Y, Wang S, Zhang F, Zhuang C (2019). Clinical features and outcomes of tetanus: a retrospective study. Infect Drug Resist.

[7] Almas T, Niaz MA, Zaidi SM (2021). The spectrum of clinical characteristics and complications of tetanus: a retrospective cross-sectional study from a developing nation. Cureus.

[8] Shahade A, De Jesus O (2024). Opisthotonus. StatPearls [Internet].

[9] Thwaites CL, Beeching NJ, Newton CR (2015). Maternal and neonatal tetanus. Lancet.

[10] Rodrigo C, Fernando D, Rajapakse S (2014). Pharmacological management of tetanus: an evidence-based review. Crit Care.

[11] Boots RJ, Lipman J, O'Callaghan J, Scott P, Fraser J (2000). The treatment of tetanus with intrathecal baclofen. Anaesth Intensive Care.

[12] (2018). Tetanus vaccines: WHO position paper, February 2017 - recommendations. Vaccine.

[13] Kakou AR, Eholie S, Ehui E (2001). Localized tetanus in Abidjan: clinical and prognostic features (1976-1997) (Article in French). Bull Soc Pathol Exot.

[14] (2024). Tetanus. https://www.who.int/news-room/fact-sheets/detail/tetanus.

[15] Cardinal PR, Henry SM, Joshi MG, Lauerman MH, Park HS (2020). Fatal necrotizing soft-tissue infection caused by Clostridium tetani in an injecting drug user: a case report. Surg Infect (Larchmt).

[16] Thwaites L (2024). Tetanus. UpToDate.

